# Podocyte, tubular epithelial-immune cell interplay in the pathogenesis of lupus nephritis

**DOI:** 10.3389/fimmu.2025.1682075

**Published:** 2025-11-25

**Authors:** Jingyi Chen, Zixiang Chen, Xiao Mou, Ke Rui, Jie Tian

**Affiliations:** 1 Department of Laboratory Medicine, Affiliated Hospital of Jiangsu University, Zhenjiang, China; 2 Department of Immunology, Jiangsu Key Laboratory of Laboratory Medicine, School of Medicine, Jiangsu University, Zhenjiang, China; 3 Department of Nuclear Medicine, Affiliated Hospital of Jiangsu University, Zhenjiang, China

**Keywords:** lupus nephritis, podocytes, tubular epithelial cells, immune cells, immune responses

## Abstract

Lupus nephritis (LN), a severe complication of systemic lupus erythematosus (SLE), is associated with increased morbidity and mortality. The pathogenesis of LN involves complex immune-mediated mechanisms that alter the biology of renal resident epithelial cells. Emerging evidence highlights the bidirectional interactions between immune cells and renal epithelial cells—including podocytes and tubular epithelial cells(TECs)—as critical contributors to disease progression. These interactions shape local immune responses, drive inflammatory injury, and disrupt renal function. However, the molecular and cellular basis of this crosstalk remains incompletely understood. Recent advances have uncovered key mechanisms underlying these interactions and identified potential therapeutic targets that may inform future treatment strategies. This review summarizes current findings on the immunological roles of renal epithelial cells in LN and discusses their relevance to the development of targeted and cell-specific therapeutic interventions.

## 1 Introduction

Systemic lupus erythematosus (SLE) is a prototypical systemic autoimmune disorder in which dysregulated immunity drives self-directed attacks on multiple tissues (1). Worldwide, its incidence ranges from 1 to 8.7 cases per 100 000 person-years and its prevalence from 8 to 180 cases per 100 000 population (2, 3).

The disease exhibits a striking female predominance, especially among women of reproductive age, with a female-to-male ratio of 6.1–13.3 : 1 (2, 3). Impaired clearance of apoptotic cells and loss of tolerance to endogenous antigens, such as double-stranded DNA (dsDNA), underlie persistent immune activation (4). Consequently, both innate and adaptive responses are amplified, driving high titers of anti-nuclear autoantibodies, formation of immune complexes (ICs), and complement activation (5). IC deposition in glomerular capillary walls provokes robust local inflammation and is a major determinant of lupus-related renal dysfunction, underscoring the kidney’s particular vulnerability in SLE (6–8).

Lupus nephritis (LN) is the most frequent and clinically devastating complication of SLE (9). It is defined by immune-complex–driven chronic inflammation that produces multi-compartmental renal injury (**Table 1**) (10). Nearly 40 % of patients develop LN within five years of an SLE diagnosis (11), and in some individuals the renal syndrome is the initial presentation that prompts recognition of underlying SLE (12, 13). LN substantially increases morbidity and mortality; 10–20 % of affected patients progress to end-stage renal disease (ESRD) within five years (14). ESRD refers to the stage of kidney disease that requires dialysis or kidney transplantation, and patients are usually accompanied by a severe symptom burden and multiple chronic conditions (MCC) (15). This disease has a particularly profound impact on patients’ quality of life (QoL) (16, 17). From the initiation of dialysis, the life expectancy of patients with ESRD is generally less than 3 years. Therefore, ESRD not only means that patients need lifelong dependence on renal replacement therapy but also brings a heavy economic and social burden to individuals and public health systems (18, 19). It is noteworthy that the prevalence and severity of LN show significant racial and ethnic differences. Large epidemiological studies have shown that the risk of disease is significantly higher in African, Asian, and Hispanic populations than in white populations (3, 9, 20–24), among which African and Asian patients also have a significantly increased risk of end-stage renal disease (25). An international inception cohort study showed that the incidence of LN in patients with systemic lupus erythematosus was 39.9% in African individuals, 49.3% in Hispanic individuals, 36.8% in Asian individuals, and 20.3% in white individuals (26). Similarly, a special study on SLE patients in the United States found that, compared with the white population, the risks of LN were higher in Black, Asian and Pacific Islander, and Hispanic populations (24). In addition, male SLE patients and those with childhood-onset disease are more likely to progress to severe renal lesions (26–34).

**Table 1:** Clinical manifestations of lupus nephritis.

Clinical features	Description	Ref.
Renal Status	The most common abnormalities in LN patients include proteinuria, microscopic hematuria (with or without red blood cell casts), renal dysfunction, nephrotic-range proteinuria or nephrotic syndrome, and hypertension.	(266–269)
Diagnosis	Renal biopsy remains the gold standard for diagnosing LN (typically not performed in patients with urinary protein excretion <500 mg/day and no significant urinary sediment abnormalities), as it enables determination of renal involvement characteristics, exclusion of alternative causes of kidney injury, identification of LN histopathological subtypes, and evaluation of disease activity and chronicity.	(240, 243, 270, 271)
Pathological Findings	Glomerular immune deposits are characterized by predominant IgG staining, together with concurrent deposition of IgA, IgM, C3, and C1q, a pattern classically described as “full-house” immunofluorescence. Immune complex deposits may be observed in mesangial, subendothelial, and subepithelial regions. Extraglomerular immune complexes deposit along tubular basement membranes, in the interstitium, and within vessels. In severe LN (typically ISN/RPS class III and IV, particularly class IV), such extraglomerular deposits are highly prevalent and widely distributed, whereas in mild LN (class I and II), they are generally infrequent, restricted, or even absent. Tubuloreticular inclusions are present in glomerular endothelial cells.	(271–274)
Histopathological Classification	The clinical manifestations of LN are not closely correlated with renal histopathological types; therefore, accurate determination of renal pathology is essential for guiding subsequent therapeutic strategies. Based on the International Society of Nephrology/Renal Pathology Society (ISN/RPS) 2004 classification system, SLE-related glomerular diseases are categorized into six types (273, 275, 276).Lupus podocytopathy and lupus-associated thrombotic microangiopathy (TMA) represent distinct pathological variants that may significantly influence treatment strategies and prognosis, and should therefore be recognized as special pathological types in LN: Class I: Minimal mesangial lupus nephritis Class II: Mesangial proliferative lupus nephritis Class III: Focal lupus nephritis Class IV: Diffuse lupus nephritis Class V: Lupus membranous nephropathy Class VI: Advanced sclerosing lupus nephritis	(273, 275, 276)

**Figure 1:**
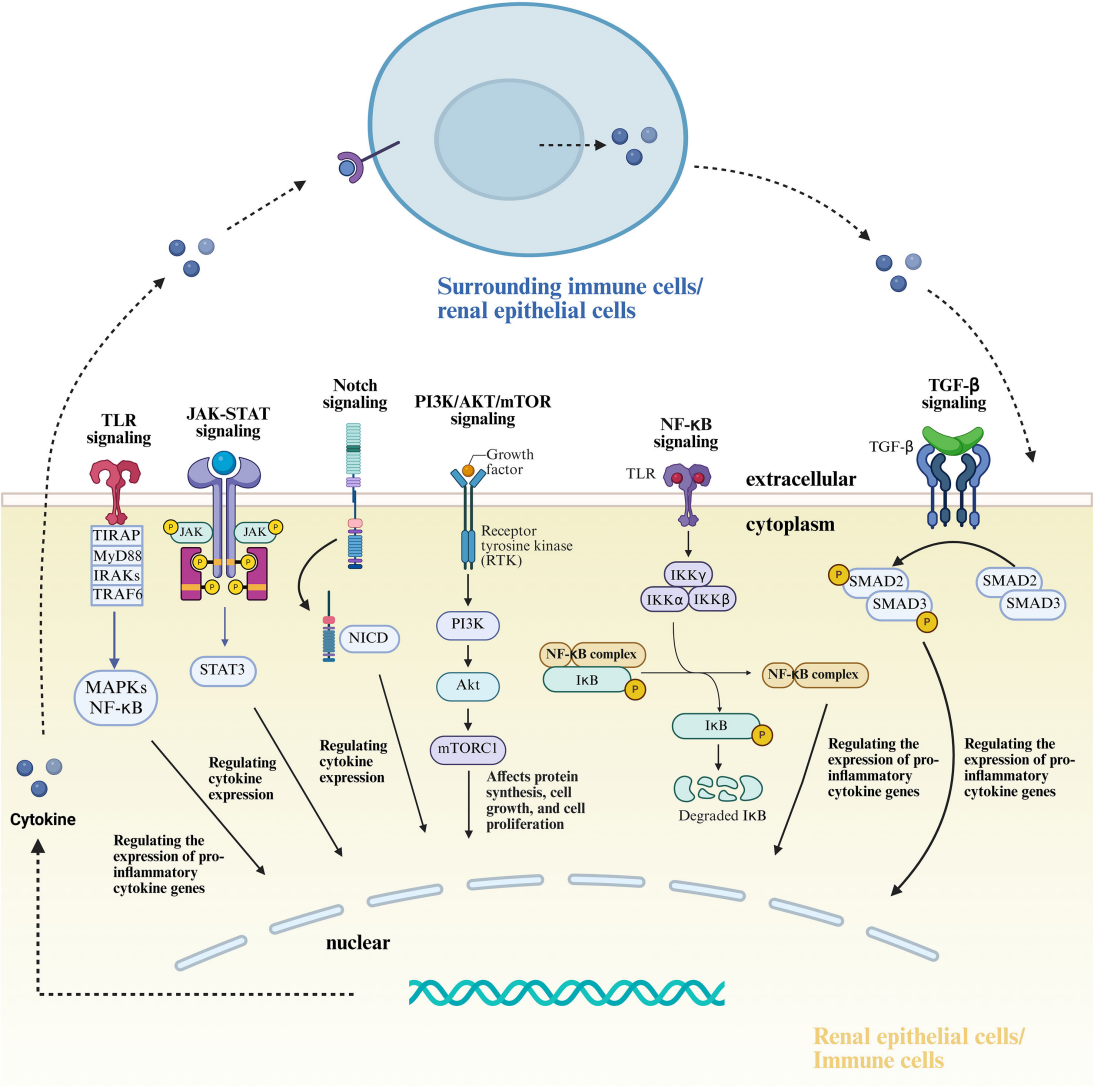
Signal pathways involved between renal epithelial cells and immune cells.

Immune-mediated renal inflammation in LN involves not only aberrant immune cell responses but also the dysfunction of resident renal cells, particularly podocytes and renal tubular epithelial cells (RTECs) (35). These two components together contribute to glomerular, tubulointerstitial, and vascular injury, ultimately driving the initiation and progression of LN (8). Podocytes, integral to the glomerular filtration barrier, express a variety of pattern recognition receptors—including Toll-like receptors (TLRs)—as well as complement-associated proteins (36, 37). Upon pathological stimulation, these receptors activate multiple intracellular pathways that lead to podocyte injury and disruption of barrier integrity.RTECs also play an active role in renal inflammation by secreting proinflammatory mediators and engaging in adaptive immune responses. For instance, anti-double-stranded DNA (anti-dsDNA) antibodies can stimulate RTECs to produce tumor necrosis factor-α (TNF-α), which enhances immune cell recruitment and triggers downstream inflammatory cascades (38). In parallel, various immune cell subsets—including neutrophils, T and B lymphocytes, dendritic cells, and macrophages—are robustly activated in LN. Through cytokine secretion, antibody production, and tissue infiltration, they exacerbate structural damage to the kidney (39). Renal epithelial cells and immune cells engage in a bidirectional regulatory interplay mediated by cytokine networks and direct cell–cell contact (40). This crosstalk cooperatively activates multiple proinflammatory signaling pathways (**Table 2**, **Figure 1**), thereby driving the immunopathological progression of LN. For instance, *in vitro* studies have shown that IL-6 secreted by podocytes acts on glomerular endothelial cells (GECs) to inhibit neutrophil recruitment, whereas neutrophils, in turn, compromise podocyte function by releasing proteolytic enzymes and other mediators (41), ultimately contributing to LN progression. This interplay amplifies renal inflammation and drives the immunopathogenesis of LN. This review highlights the complex crosstalk between renal epithelial cells and immune cells within the kidney and delineates their mechanistic roles in the development and progression of lupus nephritis.

**Table 2:** Signal pathways involved between renal epithelial cells and immune cells.

Signaling pathway	Role description	Significance	Ref.
TLR Signaling Pathway	TLR4 recognizes extracellular HMGB1, activates AP-1 and upregulates IRF3/5/7. Signaling via MyD88 and TRIF promotes NF-κB translocation, regulating pro-inflammatory cytokine gene expression.	TLRs, particularly TLR4, are widely expressed on renal epithelial and immune cells. Overactivation of TLR4 signaling correlates with SLE activity and LN progression.	(277–281)
JAK-STAT Signaling Pathway	Aberrant STAT signaling in LN is driven by JAK kinases. IL-6 and IL-21 stimulate antibody production via STAT3-dependent pathways. STAT3 activation also enhances IL-6 expression and is critical for follicular helper T-cell (Tfh) differentiation.	The JAK-STAT pathway plays a pivotal role in immune cell activation and inflammatory responses by modulating cytokine expression.	(282, 283)
Notch Signaling Pathway	Canonical Notch signaling is activated by receptor (Notch1-4) and ligand (DLL1/3/4, JAG1/2) interactions, leading to nuclear translocation of the Notch intracellular domain (NICD) to regulate target genes.	Notch signaling is evolutionarily conserved and essential for development and homeostasis. In LN, Notch1 dysregulation may influence podocyte differentiation and glomerular pathology.	(284–292)
CXCL10-CXCR3 Axis	The CXCL10-CXCR3 axis promotes Th1 cell infiltration into the kidneys, exacerbating inflammation. CXCL10 binding to CXCR3 activates downstream pathways, driving Th1 chemotaxis and activation.	This axis is a key mediator of CXCR3^+^T-cell renal infiltration, contributing to LN progression.	(293, 294)
PI3K/Akt/mTOR Signaling Pathway	mTOR, a serine/threonine kinase, regulates cell proliferation, metabolism, and cell cycle progression (G1 to S phase), thereby modulating T- and B-cell activation.	PI3K/Akt/mTOR activation is critical for anti-apoptotic signaling, proliferation, and inflammatory cytokine production.	(295–298)
NLRP3 Signaling Pathway	The NLRP3 in of NACHT, LRR, and PYD domains, is implicated in LN pathogenesis.	NLRP3 signaling is central to innate immunity and is closely linked to inflammatory and immune responses in LN.	(299–301)
NF-κB Signaling Pathway	In homeostasis, NF-κB is sequestered in the cytoplasm by IκB. Upon activation, IκB is phosphorylated and degraded by IKK, enabling NF-κB nuclear translocation to initiate transcription of pro-inflammatory genes (e.g., TNF-α, IL-6).	NF-κB is a master regulator of inflammatory mediators, driving cytokine production in LN.	(279, 302, 303)
TGF-β Signaling Pathway	TGF-β binds to TGF-βR2, recruits TGF-βR1, and induces Smad2/3 phosphorylation, activating downstream transcriptional programs.	The TGF-β pathway is a core driver of renal fibrosis in LN, exacerbated by pro-inflammatory cytokines.	(304, 305)
B7-1/CD28 Signaling Pathway	B7-1 (CD80) binding to CD28 provides co-stimulatory signals, promoting T-cell proliferation, Th1/Th2 differentiation, and antibody production.	Hyperactivation of B7-1/CD28 signaling may trigger autoimmune responses, contributing to LN pathogenesis.	(306–310)

Renal epithelial cells/immune cells regulate transcription through various signaling pathways, and the cytokines produced can affect immune cells/renal epithelial cells.

## 2 Basic structure and function of podocytes

**Figure 2:**
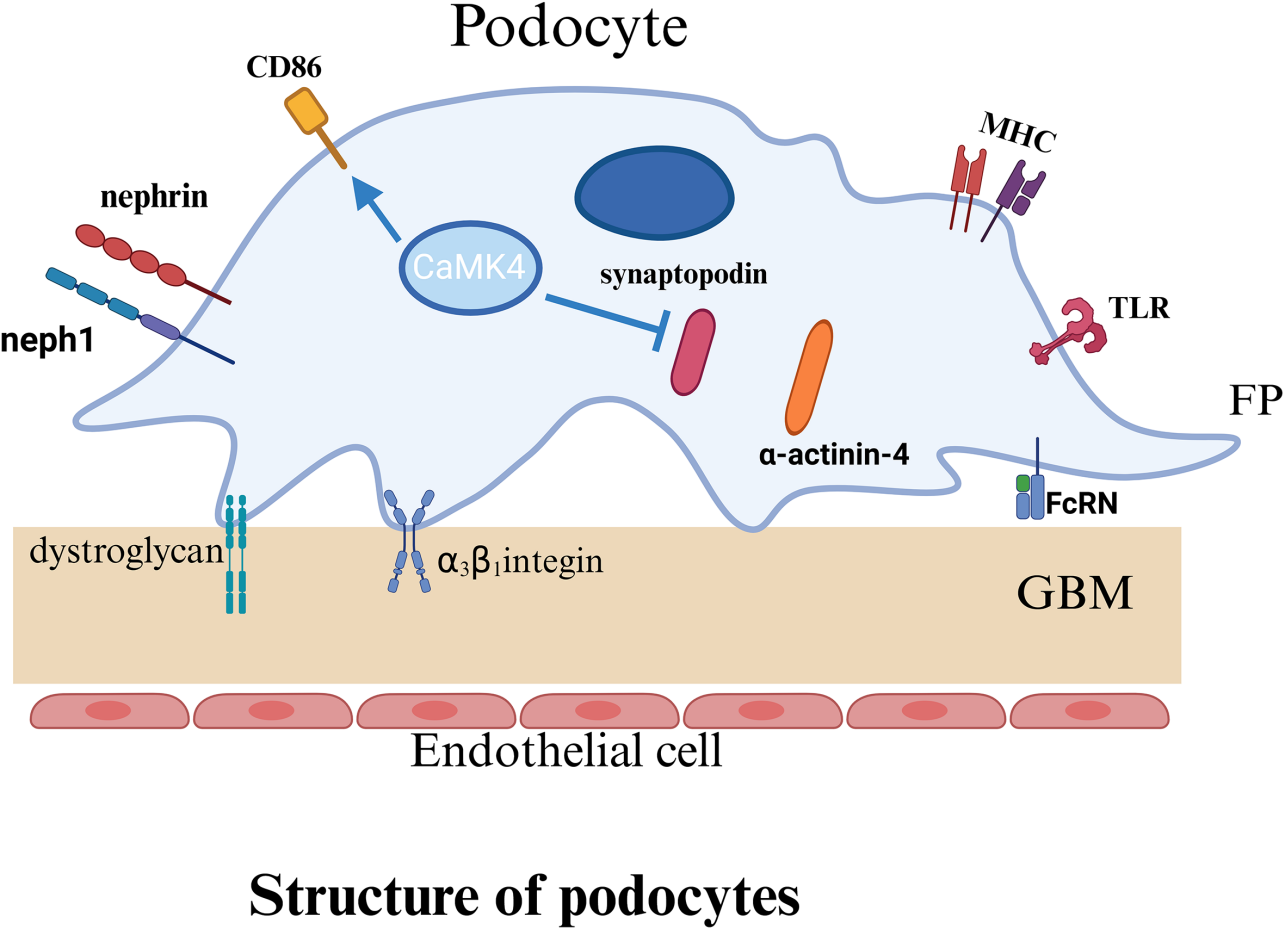
Podocyte basic structure.

Podocytes are an essential component of the glomerular filtration barrier, and their highly specialized morphology (see **Figure 2**) is critical for preserving filtration function. Interdigitating foot processes of podocytes form the slit diaphragm (SLD) (42, 43), a specialized junctional structure composed primarily of nephrin and podocin. These proteins, in conjunction with the actin cytoskeleton and adhesion molecules, establish a stable network that preserves the structural integrity of the filtration barrier (44, 45). Studies have shown that aberrant IgG glycosylation in patients with SLE contributes to podocyte injury in LN, leading to cytoskeletal remodeling, impaired motility, and reduced nephrin production (46). These alterations accelerate the progression of LN (46). Nephrin, a transmembrane immunoglobulin-like adhesion molecule (47), constitutes the structural backbone of the SLD and is essential for maintaining the mechanical cohesion and selective permeability between adjacent foot processes (48). Podocin, another key transmembrane protein, interacts with nephrin to stabilize its membrane localization (49) and link it to intracellular signaling pathways, thereby supporting podocyte architecture and function (50). Deficiency or dysfunction of either protein disrupts the integrity of the filtration barrier and leads to the development of proteinuria. Relevant studies have demonstrated that podocyte-associated proteins, such as nephrin, podocin, and podocalyxin, serve as important indicators for assessing podocyte injury in human glomerular diseases (51). Accordingly, the loss or dysfunction of nephrin and podocin not only disrupts the structural integrity of the slit diaphragm but also directly compromises the filtration barrier, ultimately leading to proteinuria.

The structural integrity of podocyte foot processes is primarily supported by actin, a highly dynamic cytoskeletal element essential for maintaining cellular morphology, adhesion, and motility (52). The reorganization of actin filaments is tightly regulated by synaptopodin (53), an actin-associated protein that reinforces cytoskeletal architecture and modulates podocyte migration. Synaptopodin interacts with α-actinin-4 to stabilize the structure and morphology of foot processes (54). It also protects the small GTPase RhoA from proteasomal degradation, thereby promoting stress fiber formation and regulating cytoskeletal tension and motility (55). For instance, studies in lupus-prone mouse models have revealed that synaptopodin expression is upregulated during podocyte apoptosis (56). Therefore, synaptopodin expression is closely associated with podocyte injury and may contribute to the progression of LN (56). In addition, podocytes adhere to the glomerular basement membrane (GBM) through integrins (such as α3β1) and α-/β-dystroglycans, which anchor the cells to the GBM and mediate outside-in signaling critical for maintaining filtration barrier stability (42, 53).

In LN, podocytes serve not only as structural targets but also as active participants in disease pathogenesis. Within the inflammatory milieu, cytokines and immune complexes generated by immune activation trigger reorganization of the actin cytoskeleton, effacement of foot processes, and disruption of the slit diaphragm, thereby aggravating proteinuria and impairing renal function. Podocytes in LN upregulate immune-related molecules such as TLRs, complement receptors, and MHC class II (57–59), and aberrantly express the neonatal Fc receptor (FcRn) under inflammatory stress (60–62), which facilitates immune complex transcytosis and deposition. Moreover, podocytes produce a range of cytokines (57), contributing to local immune modulation and amplifying inflammatory responses, while also possessing potential immunoregulatory functions. Importantly, podocytes can present antigens (63) and support the activation of T and B lymphocytes, thereby enhancing autoantibody production and perpetuating tissue injury (47, 64). The integrity of podocyte structure—dependent on cytoskeletal organization, slit diaphragm maintenance, and anchorage to the GBM—is disrupted by immune-mediated cytoskeletal remodeling, which is considered a central mechanism of podocyte injury in LN. This process is driven by a convergence of inflammatory insults, genetic predisposition, toxic exposures, and metabolic disturbances, ultimately leading to filtration barrier breakdown and disease progression (65).

A schematic illustration of the basic structure of the podocyte indicates the structure and important proteins of the podocyte. P, podocyte; FP, foot process; GBM, glomerular basement membrane; TLR, Toll like receptors; MHC, major histocompatibility complex; FcRn, Neonatal Fc receptor.

## 3 Crosstalk between podocytes and innate immune cells

**Figure 3:**
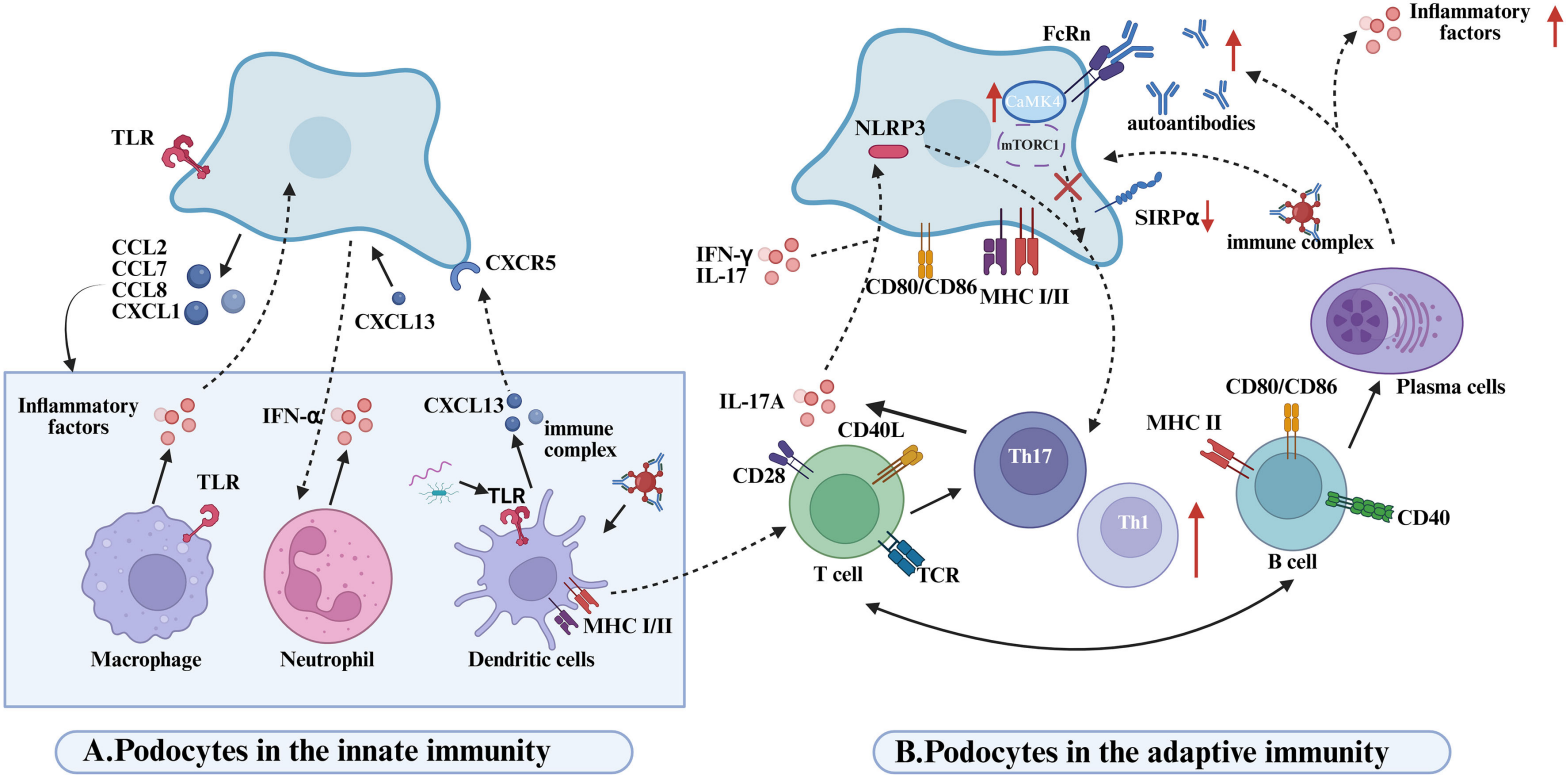
Disease mechanisms mediated by podocyte-immune cell interactions in lupus nephritis. **(A)** Podpcytes in the innate immunity. Podocytes recruit innate immune cells (macrophages, neutrophils, DCs) via chemokine secretion (CCL2/7/8, CXCL1), amplifying inflammation. Macrophage-derived proinflammatory cytokines drive proteinuria in glomerular diseases. TLRs on macrophages/DCs recognize PAMPs, activating innate immunity. Neutrophil infiltration and IFN-α release exacerbate renal inflammation in LN. CXCL13-stimulated podocyte media triggers neutrophil activation and local inflammation. DCs produce CXCL13, binding podocyte CXCR5 to activate ERK signaling and enhance proinflammatory secretion, worsening LN. DCs phagocytose apoptotic/immune complexes to promote Th17 differentiation.Th17-derived IL-17A directly damages podocytes and induces NLRP3 inflammasome/IL-1β release, sustaining inflammation. pDCs detect microbial/eukaryotic nucleic acids via TLR9, driving IFN-I production and systemic immune activation through costimulatory upregulation. **(B)** Podpcytes in the adaptive immunity. Podocytes activate T cells via MHC I/II and costimulatory molecule expression; CD80 enhances T-cell proliferation/aggregation. LN podocytes exhibit SIRPα downregulation, promoting Syk phosphorylation to boost antigen presentation and T-cell responses. IFN-α/γ and IL-17 modulate podocyte antigen presentation by targeting SIRPα.LN IgG enters podocytes via FcRn, inducing CaMK4-CD86 upregulation to amplify T-cell costimulation in a self-reinforcing loop.NLRP3 activation in injured podocytes biases Th17/Th1 differentiation; Th17-derived IL-17A disrupts podocyte cytoskeleton and triggers inflammation. Podocyte mTORC1 activation drives glomerulopathy via endothelial crosstalk, while T-cell mTORC1 hyperactivity expands Th1/Th17/DN T cells and suppresses Tregs. B cells directly damage kidneys via autoantibodies; B1 cells accumulate in LN kidneys, differentiating into autoantibody/cytokine-producing plasma cells.

Emerging evidence indicates that, beyond their structural role in preserving glomerular integrity, podocytes actively engage in reciprocal interactions with innate immune cells—including macrophages, neutrophils, and dendritic cells—which collectively orchestrate local inflammation and contribute to the pathogenesis of LN (**Figure 3A**). In this context, podocytes function not only as passive targets of immune-mediated injury but also as active regulators of renal inflammation. Upon stimulation, podocytes secrete various chemokines, such as CCL2, CXCL1, and CXCL13, which recruit monocytes (66), neutrophils (67), and dendritic cells (67) into the glomerulus, thereby amplifying inflammatory responses and promoting renal damage. Of note, dendritic cells represent a major source of CXCL13 (68), which binds to CXCR5 receptors expressed on podocytes and activates the ERK signaling pathway, subsequently inducing the expression of adhesion molecules ICAM-1 and VCAM-1 (69). Through chemotactic gradients, these chemokines facilitate the recruitment of innate immune cells, such as macrophages and neutrophils, to sites of inflammation, reinforcing the local immune response. Notably, blockade of CCL2 or its receptor CCR2 significantly reduces monocyte/macrophage recruitment to the kidney and alleviates glomerular and interstitial injury (70, 71). The infiltrating innate immune cells perform distinct immunological functions within the renal microenvironment, ultimately shaping the progression of LN pathology.

Recruited macrophages infiltrate extensively into LN lesions and release pro-inflammatory cytokines such as TNF-α and IL-1β, which downregulate the expression of critical podocyte structural proteins, including nephrin (72). This disruption compromises the glomerular filtration barrier, ultimately resulting in proteinuria. The formation of a positive feedback loop between activated podocytes and infiltrating macrophages plays a pivotal role in sustaining renal inflammation during LN. Moreover, the synergistic activation of Toll-like receptors TLR2 and TLR4 further amplifies this inflammatory circuit. These receptors are broadly expressed on both podocytes and macrophages and can recognize pathogen-associated molecular patterns (PAMPs) as well as endogenous damage-associated molecular patterns (DAMPs), leading to downstream signal transduction and the production of inflammatory mediators such as TNF-α and IL-1β (73). TLR-mediated co-activation not only intensifies the pro-inflammatory capacity of macrophages but also enhances the immunological responsiveness of podocytes, thereby exacerbating glomerular inflammation and accelerating LN progression (74). In parallel, neutrophils are also markedly recruited into the renal tissues of LN patients (75), where they secrete interferon-α (IFN-α), reactive oxygen species (ROS), and proteolytic enzymes that contribute to tissue injury (41). Neutrophils undergoing cell death release neutrophil extracellular traps (NETs)—web-like structures composed of decondensed DNA and antimicrobial proteins—which can bind autoantigens and trigger immune activation. This process further amplifies the inflammatory cascade and promotes the pathophysiological progression of LN (41).

In macrophage-mediated podocyte injury, the activation state of macrophages is a key determinant of renal inflammatory outcomes. Macrophage phenotypes are largely shaped by cues from the local microenvironment (76) and are broadly categorized into pro-inflammatory M1 (classically activated) and anti-inflammatory M2 (alternatively activated) subsets (77). In LN, a disruption in the M1/M2 balance is closely linked to disease activity (78). The active phase of LN is typically dominated by M1 macrophages (79), which secrete high levels of inflammatory mediators such as TNF-α and IL-1β.These cytokines downregulate the expression of essential podocyte structural proteins, disrupt the glomerular filtration barrier, and exacerbate both proteinuria and tissue inflammation. In contrast, during disease remission, the prevalence of M2 macrophages increases (80). These cells release anti-inflammatory cytokines and tissue-reparative factors, which contribute to podocyte protection and restoration of glomerular barrier integrity. Transcriptomic profiling has revealed a dynamic shift of macrophages from an M1 to M2 phenotype during LN progression, accompanied by suppressed expression of pro-inflammatory genes (81). This phenotypic transition exerts protective effects on podocytes by attenuating inflammatory damage. Collectively, macrophage polarization from a pro-inflammatory to an anti-inflammatory state appears to mitigate podocyte injury (72) and may serve as an intrinsic regulatory mechanism during disease resolution (72, 80). Consequently, therapeutic strategies aimed at promoting M2 polarization have gained attention as a promising approach to slowing LN progression and preserving podocyte integrity (82).

In addition to interacting with macrophages and neutrophils, podocytes engage in dynamic and bidirectional crosstalk with DCs, actively shaping the local immune microenvironment in LN (83). DCs recruited to the kidney play a non-redundant role in LN immunopathogenesis (84, 85). Experimental studies have demonstrated that, despite persistent glomerular deposition of immune complexes, either genetic deficiency of Fcγ receptors or selective depletion of DCs can effectively prevent T cell activation and leukocyte infiltration in renal tissues. DCs contribute to the differentiation of Th17 cells by phagocytosing apoptotic bodies or immune complexes (83). In turn, Th17-derived IL-17A not only exerts direct cytotoxic effects on podocytes but also induces NLRP3 inflammasome activation and promotes IL-1β release, thereby amplifying the local inflammatory cascade (4). Furthermore, DCs activate autoreactive T cells through self-antigen presentation, which facilitates B cell-driven production of pathogenic autoantibodies, such as anti-dsDNA antibodies. These autoantibodies can form immune complexes with podocyte surface antigens, including α-actinin, and upon deposition in the glomeruli, activate the complement cascade. This leads to cytoskeletal rearrangement and disruption of the slit diaphragm architecture in podocytes, thereby exacerbating glomerular injury (86).

## 4 Crosstalk between podocytes and adaptive immune cells

Podocytes, once considered mere passive targets in LN, are now recognized as active participants in immune modulation. Through direct interaction with T and B lymphocytes, they shape adaptive immune responses and contribute to glomerular inflammation and injury (**Figure 3B**).

### 4.1 Podocytes and T lymphocytes

In the pathogenesis of LN, interactions between T cells and podocytes play a pivotal role in disrupting the glomerular filtration barrier and propagating inflammation. Inflammatory mediators released by autoreactive T cells amplify local renal immune responses, while certain autoantibodies—some of which cross-react with podocyte antigens such as α-actinin—further exacerbate renal tissue injury (87, 88). Increasing evidence highlights the crucial and diverse roles of dysregulated helper T (Th) cell subsets in mediating podocyte damage. Based on their cytokine profiles, Th cells are categorized into distinct functional subsets (89), each contributing differentially to the pathological processes involved in podocyte injury. Among them, Th17 cells predominantly produce IL-17A and IL-17F, whose receptors are broadly expressed across various renal cell types, including podocytes. These cytokines activate pro-inflammatory and pro-fibrotic pathways, promoting the release of cytokines and chemokines that sustain chronic inflammation and maladaptive tissue repair, ultimately resulting in fibrosis and disruption of renal architecture (90). IL-17A, in particular, has been shown to alter podocyte morphology and induce structural injury (91). Moreover, in LN, activation of the Th17 lineage and elevated IL-17 levels are associated with podocyte apoptosis, cytoskeletal rearrangement, foot process effacement, increased motility, reduced expression of homeostatic proteins, and heightened oxidative stress, along with activation of inflammasomes and caspase cascades within podocytes (92–94). Importantly, IL-17A can also engage surface receptors on podocytes to trigger NLRP3 inflammasome activation and subsequent IL-1β release, thereby amplifying local inflammatory responses (4, 95). The activation of NLRP3 inflammasomes within podocytes not only contributes to structural cell damage but may also reshape the local immune milieu, influencing adaptive immune responses. Hutton et al. (96) proposed that NLRP3 activation in podocytes facilitates the skewing of helper T cells toward the Th17 phenotype, further promoting podocyte injury in LN. Beyond Th17 involvement, Th2 cells have also been implicated in podocyte damage in specific LN subtypes.Th2-driven immune responses are thought to facilitate subepithelial immune complex deposition (97–99), leading to complement activation and formation of the membrane attack complex (C5b-9), which injures renal epithelial cells. Additionally, both inflammasomes and mTORC1 signaling pathways have been shown to regulate Th cell differentiation, thereby contributing to the immunopathological landscape of LN (100, 101). As a key component of the innate immune system, the NLRP3 inflammasome can be activated and assembled in response to a wide array of exogenous and endogenous danger signals (102).

Accumulating evidence has demonstrated that depletion of T cells or blockade of T cell activation significantly alleviates LN progression in lupus-prone mice (103, 104). Notably, podocytes themselves are not merely passive targets but active participants in modulating T cell responses. They express both MHC class I and class II molecules (63, 105), thereby possessing antigen-presenting capacity and directly contributing to the activation of CD4^+^ and CD8^+^T cells during LN pathogenesis. Conditional deletion of MHC class II in podocytes markedly reduces CD4^+^T cell activation, highlighting their immunoregulatory potential (63). In parallel, CD8^+^T cells recognizing disease-relevant antigens presented by podocytes can exert cytotoxic effects, contributing to crescent formation within glomeruli (106). Crescents—characterized by the proliferation of parietal epithelial cells and infiltration of inflammatory cells in Bowman’s space—are histological hallmarks of severe glomerular injury and are associated with poor clinical outcomes (106, 107). Furthermore, podocytes can enhance T cell activation through the expression of costimulatory molecules such as CD80 (B7-1) and CD86 (B7-2). Inflammatory stimuli, such as TLR activation, upregulate CD80 expression on podocytes (108, 109), which facilitates T cell proliferation and accumulation within renal tissue (81). Beyond its immunostimulatory role, CD80 also compromises slit diaphragm integrity, exacerbating podocyte injury and impairing glomerular filtration barrier function (110). CD80 and CD86 interact with T cell receptors CD28 or CTLA-4 to deliver activating or inhibitory signals, thereby fine-tuning the strength and outcome of T cell responses (111, 112). Elevated B7–1 expression has been observed in podocytes from LN patients, where its activation induces redistribution of key slit diaphragm proteins such as nephrin and podocin, correlating strongly with proteinuria severity (109). Moreover, podocytes are capable of internalizing immune complexes, particularly IgG, and can activate infiltrating T cells via cross-presentation mechanisms. This activation triggers the release of proinflammatory cytokines that contribute to podocyte dysfunction and apoptosis (105). FcRn plays a crucial role in IgG transcytosis in podocytes, facilitating antigen sampling and immune modulation. IgG internalization via FcRn induces the expression of CaMK4 (113), a key mediator downstream of TCR signaling that is aberrantly activated in T cells from SLE patients and contributes to immune dysregulation (36). In podocytes, CaMK4 upregulates CD86 expression (113), amplifying costimulatory signaling and further exacerbating local inflammation and tissue injury (62).

In addition to intrinsic immunogenic properties, the ability of podocytes to activate T cells is further modulated by the inflammatory milieu. Proinflammatory cytokines such as IFN-γ and IL-17 have been shown to enhance the antigen-presenting capacity of podocytes, thereby promoting antigen-specific T cell activation and contributing to podocyte injury and renal inflammation (114). Mechanistically, signal regulatory protein alpha (SIRPα), expressed in podocytes, functions as a negative regulator of antigen presentation. It exerts its inhibitory effect by suppressing phosphorylation of spleen tyrosine kinase (Syk), a key molecule in downstream immune signaling (114). However, proinflammatory cytokines including IFN-α, IFN-γ, and IL-17 can downregulate SIRPα expression, thereby relieving this inhibitory checkpoint and augmenting the ability of podocytes to activate T cells (115–120). Supporting this, exposure of podocytes to LN patient serum has been shown to induce T cell proliferation and increased IFN-γ production, suggesting that under inflammatory conditions, podocytes can acquire dendritic cell-like immunostimulatory properties (121). Notably, IFN-γ not only promotes T cell activation but also modulates podocyte function in a feedback manner by suppressing SIRPα expression, further amplifying local immune activation.

### 4.2 Podocytes and B lymphocytes

B lymphocytes play a pivotal role in the pathogenesis of LN (7, 122), primarily through the production of autoantibodies and pro-inflammatory cytokines that contribute to renal injury, particularly targeting podocytes (123). In LN, aberrantly activated B cells differentiate into plasma cells that secrete pathogenic autoantibodies, including anti-dsDNA antibodies (124).

The accumulation of autoantibodies within the kidney is a critical contributor to renal inflammation and dysfunction in lupus nephritis (125, 126). These autoantibodies directly target podocyte surface antigens, such as α-actinin-4, inducing cytoskeletal remodeling that leads to podocyte injury (124), dedifferentiation, apoptosis, or detachment (114). Concurrently, antibody binding activates the complement cascade, compromising the integrity of the podocyte filtration barrier and resulting in proteinuria (58, 88). ICs formed by the interaction of antibodies with glomerular basement membrane or podocyte antigens deposit in close proximity to podocytes, acting as principal mediators of podocyte-directed immune injury (127) and thereby impairing glomerular filtration function (128). Moreover, anti-nuclear antibodies associate with nucleosomes released from apoptotic cells to generate proinflammatory complexes localized within the glomerulus (129). Notably, anti-dsDNA antibodies are enriched in renal tissues compared to systemic circulation and can cross-react with podocyte α-actinin, disrupting podocyte stability (86). Importantly, podocytes actively participate in antibody-mediated pathology rather than remaining passive targets. Upregulation of Fc receptors on their surface, particularly FcRn, facilitates IgG uptake (130). Internalized IgG triggers upregulation of CaMK4, which modulates downstream signaling pathways including RhoA. This signaling enhances podocyte motility while suppressing the expression of key slit diaphragm proteins such as nephrin and synaptopodin (62, 131, 132). Collectively, these alterations undermine podocyte structural integrity and barrier function, thereby exacerbating proteinuria. Furthermore, in lupus nephritis patients, IgG is transcytosed into podocytes via FcRn (133), where it not only activates CaMK4 signaling (62, 134) but also induces CD86 expression (113). This upregulation of CD86 enhances podocyte capacity to form immunological synapses with T cells, amplifying local immune activation. Clinical studies further reveal that aberrant glycosylation of IgG in the sera of patients is closely linked to podocyte injury. Such glycosylation-modified IgGs, primarily produced by B cells, exacerbate podocyte dysfunction and contribute to disease progression (57, 62).

Podocyte injury mediated by B cells is not solely dependent on antibodies but can be exacerbated through synergistic interactions with T cells. Specifically, T cells can increase the survival, differentiation, and antibody production of autoreactive B cells (135) and promote inflammation and tissue damage through cytokine secretion. Although podocytes are not conventional antigen-presenting cells, they express MHC class II and costimulatory molecules in lupus nephritis, enabling them to activate CD4^+^T cells (83, 136). Through such crosstalk with T cells, podocytes can indirectly modulate B cell activation and subsequent antibody production. Dysregulation of T cell function disrupts peripheral tolerance, thereby promoting B cell activation and autoantibody generation. These autoantibodies can either directly target renal structural components or form immune complexes that deposit within the glomeruli, triggering local inflammation and tissue damage (137, 138).

## 5 Renal tubular epithelial cells

Proximal tubular epithelial cells (PTECs), a metabolically active and highly polarized subset of RTECs, play a central role in maintaining renal function. Responsible for reabsorbing approximately 80% of the glomerular filtrate—including glucose, electrolytes, and amino acids—PTECs are enriched in mitochondria and exhibit significantly higher metabolic demands compared to other renal cell types (139). In addition, they express multiple iron-handling proteins, such as transferrin receptor 1 (TfR1) and divalent metal transporter 1 (DMT1), and contribute to iron homeostasis via active endocytic pathways (140).

While RTECs have traditionally been recognized for their role in solute transport, growing evidence highlights their active involvement in the immunopathogenesis of renal parenchymal diseases (141, 142), particularly in mediating inflammation and fibrosis (143). In LN, the structural and metabolic features of RTECs render them particularly susceptible to immune-mediated injury. Tubulointerstitial fibrosis is a major contributor to LN progression toward ESRD (144), often associated with PTEC-derived pro-inflammatory cytokines that activate immune cells and initiate epithelial-to-mesenchymal transition (EMT) (145). Upon injury, RTECs acquire an inflammatory phenotype characterized by the production of cytokines such as CSF-1, CCL2, IL-6, TNF-α, IL-1β, and IL-18. These mediators amplify immune responses through autocrine signaling and promote leukocyte recruitment by secreting chemokines including CCL2, CCL5, and CXCL8, thereby exacerbating tubulointerstitial inflammation and tissue damage (146). Notably, deposition of immune complexes along the tubular basement membrane—often accompanied by immune cell infiltration and interstitial fibrosis—is a common pathological feature in LN (147), underscoring the immunoregulatory role of RTECs in disease progression.

### 5.1 Crosstalk between renal tubular epithelial cells and innate immune cells

**Figure 4:**
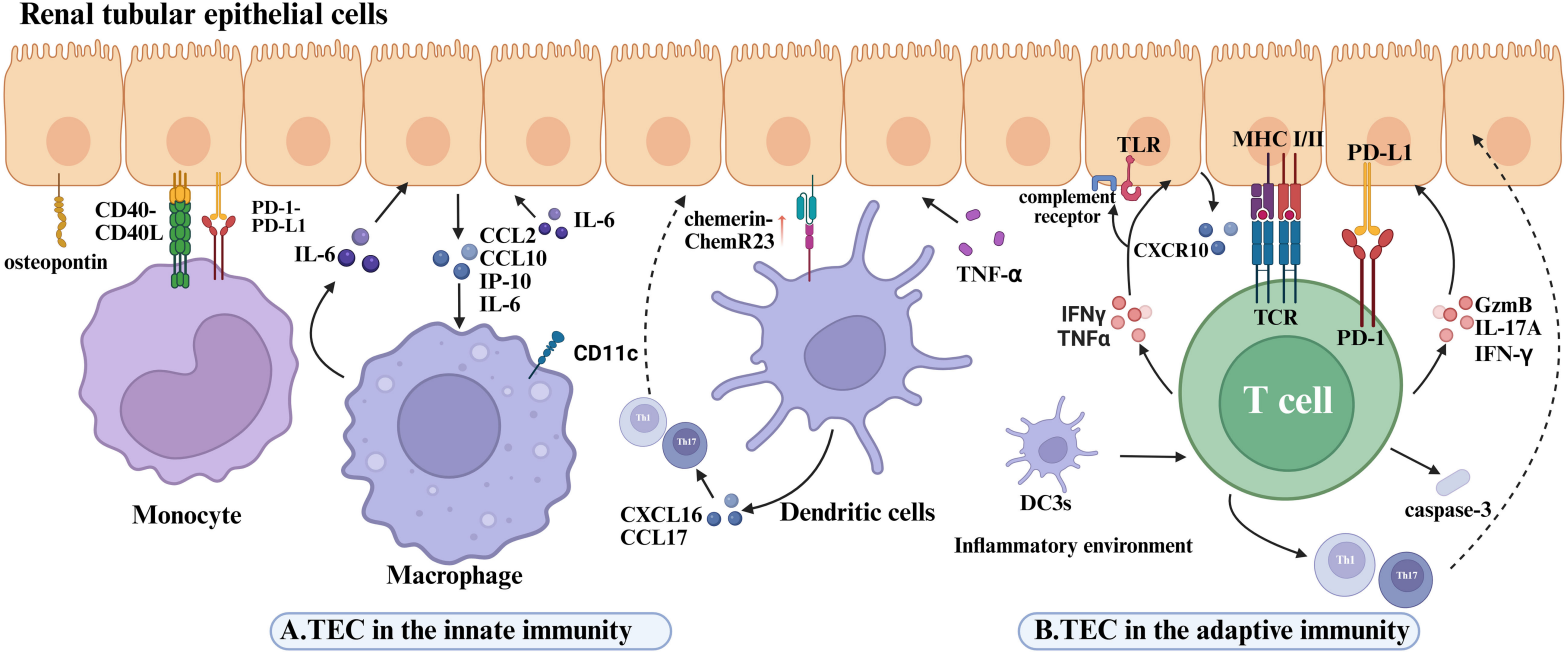
Disease mechanisms mediated by the interaction of renal tubular epithelial cells with immune cells in lupus nephritis. **(A)** The role of renal tubular epithelial cells in innate immunity. OPN expressed on RTECs is associated with monocyte infiltration and tubular injury. In LN, monocytes interact with RTECs through the CD40–CD40L axis to activate inflammatory pathways, while PD-L1 on RTECs suppresses excessive monocyte activation via PD-1. IL-22 stimulation upregulates CCL2, CXCL10, and pSTAT3 in RTECs. Macrophages are recruited to the tubulointerstitial compartment by CCL2 and CXCL10 secreted by TECs, and their released IL-6 aggravates TEC detachment and apoptosis by inducing fibronectin expression. RTECs recruit pDCs via TNF-α-induced chemerin-ChemR23 axis in LN. The tubulointerstitial DC3 subset expresses high levels of CXCL16 and CCL17, thereby exacerbating RTEC inflammatory injury. **(B)** The role of renal tubular epithelial cells in adaptive immunity. IFN-γ and TNF-α stimulation upregulate CXCL10 and CXCR3 expression in RTECs. In LN kidneys, damaged iPTECs overexpress proinflammatory mediators, promoting the recruitment of blood-derived DC3 cells to renal tissue. Renal DC3 cells are reprogrammed toward a proinflammatory phenotype within the milieu of tubular injury, driving Th1/Th17 adaptive immune responses and worsening RTEC inflammation. Injured PTECs upregulate MHC class II and costimulatory molecules and activate CD4^+^T cells through antigen presentation. RTECs also activate CD8^+^T cells via cross-presentation, inducing the release of GzmB, IL-17A, and IFN-γ. Direct contact between CD8^+^T cells and RTECs enhances caspase-3–mediated apoptosis and tubulointerstitial inflammation. Overexpression of PD-L1 on RTECs inhibits T-cell activation through PD-1, establishing an immunosuppressive microenvironment that counterbalances inflammatory injury.

RTECs play an active role in recruiting and activating innate immune cells, thereby amplifying inflammatory responses within the kidney (**Figure 4A**). Monocyte chemoattractant protein-1 (MCP-1/CCL2) is a well-established chemokine critical for monocyte recruitment in various inflammatory settings (71). In LN, MCP-1 expression is predominantly localized to the renal tubules rather than the glomeruli (148, 149), and its levels closely correlate with macrophage and T cell infiltration, thereby promoting local inflammation. Osteopontin (OPN), an inflammation-associated glycoprotein abundantly expressed by RTECs in both proximal and distal tubules, exhibits a positive correlation with the extent of monocyte/macrophage infiltration and severity of renal injury. Upregulated OPN expression has been documented across multiple glomerulonephritis models, where it facilitates monocyte adhesion and migration into the tubulointerstitial compartment through interaction with the CD44 receptor (149–151). Both clinical and experimental studies confirm that elevated OPN expression associates with increased tubular monocyte infiltration and tubular damage (152, 153), with significantly higher OPN levels observed in LN patients and murine models (154, 155). Moreover, upon stimulation by proinflammatory cytokines such as TNF-α, RTECs secrete chemerin, which recruits plasmacytoid dendritic cells (pDCs) to sites of inflammation via the ChemR23 receptor, thereby promoting local immune activation (156). RTECs also produce various chemokines that enhance dendritic cell recruitment and accumulation at lesion sites (157). Of particular interest is the infiltration of a distinct myeloid dendritic cell subset—type 3 conventional dendritic cells (DC3s)—within the renal tubulointerstitium. These DC3s are believed to act as intermediaries bridging interactions between intrinsic proximal tubular epithelial cells (iPTECs) and T cells (191). DC3s display high expression of costimulatory molecules such as CD86 and CD40, as well as chemokines including CXCL16 and CCL17, endowing them with potent antigen-presenting capacity. Consequently, they promote T cell polarization toward Th1 and Th17 phenotypes, thereby amplifying local inflammatory responses and ultimately contributing to progressive renal parenchymal injury (158). Beyond classical chemokines, recent evidence indicates that RTECs can indirectly modulate immune cell chemotaxis and activation in response to cytokine stimuli. For example, interleukin-22 (IL-22) has been shown to facilitate macrophage infiltration into the kidney in LN, indirectly exacerbating tubular epithelial cell injury and disease progression (159). *In vitro* studies demonstrate that recombinant IL-22 stimulation of primary murine renal tubular epithelial cells upregulates the expression of CCL2, CXCL10, and phosphorylated STAT3. Conditioned media from these cells exhibit potent macrophage chemotactic activity, underscoring the role of IL-22 in mediating RTEC-driven recruitment and activation of innate immune cells (159). Furthermore, tubular epithelial cells secrete additional chemokines such as IL-34 (160), CCL2, and CX3CL1 (161–163), which act synergistically to regulate macrophage recruitment and activation within the renal microenvironment.

Recruited innate immune cells and RTECs engage in bidirectional interactions through both direct contact and soluble mediators, forming an activation loop that perpetuates inflammation and initiates fibrotic processes. Specifically, RTECs interact with monocytes via surface costimulatory molecules such as CD40–CD40L, triggering intrinsic inflammatory signaling pathways within RTECs (164). This crosstalk not only amplifies the local inflammatory milieu but also contributes to tubulointerstitial fibrosis, underpinning the chronic progression of LN. As inflammation persists, RTECs continuously secrete chemokines including MCP-1 and OPN, which enhance the recruitment and accumulation of monocytes, macrophages, and other immune cells at sites of injury. Recruited macrophages, in turn, release IL-6, inducing TECs to upregulate fibronectin expression, thereby accelerating tubular epithelial cell detachment and death (165). This establishes a self-perpetuating positive feedback loop that exacerbates inflammation. Under these inflammatory conditions, TECs also become a primary source of colony-stimulating factor-1 (CSF-1) (166), which promotes macrophage survival, differentiation, and functional polarization, further driving both immune-dependent and independent kidney damage in LN (167). Beyond soluble factors, recent studies highlight that RTECs release functional exosomes to mediate intercellular communication with innate immune cells (168, 169). Zhang et al. (170) demonstrated that activated macrophages secrete exosomes enriched with miR-155, which upon uptake by tubular epithelial cells, target suppressor of cytokine signaling 1 (SOCS-1), a negative regulator of NF-κB signaling, thereby amplifying inflammation and aggravating TEC injury (170). Dual-luciferase reporter assays confirmed SOCS-1 as a direct miR-155 target in these cells (170). Furthermore, *in vivo* administration of miR-155-rich exosomes into renal tissue significantly worsened tubular injury.

Together, these findings underscore that in LN, RTECs orchestrate immune cell recruitment through multiple chemokines and engage in complex, bidirectional signaling with innate immune cells via costimulatory molecules, exosomes, and pro-inflammatory mediators, collectively amplifying inflammation and promoting tissue damage.

### 5.2 Crosstalk between renal tubular epithelial cells and adaptive immune cells

RTECs, particularly PTECs, are recognized as non-professional antigen-presenting cells (APCs) (171), capable of presenting antigens to CD4^+^T cells via MHC-II–restricted pathways (172). Under physiological conditions, MHC-II expression on PTECs is minimal (172–174); however, it is markedly upregulated in inflamed renal tissues (173, 175–177), along with increased expression of costimulatory molecules such as CD80 and CD86 (178–180). These changes collectively facilitate CD4^+^T cell activation and amplify local immune responses. Additionally, RTECs possess the machinery for antigen cross-presentation, enabling them to process internalized soluble antigens for presentation via MHC-I, thereby activating CD8^+^T cells and inducing their secretion of IFN-γ, IL-17A, and granzyme B (GzmB).These cytotoxic mediators contribute to RTEC apoptosis and drive tubulointerstitial inflammation (171). In LN murine models, direct contact between CD8^+^T cells and RTECs has been associated with increased epithelial cell apoptosis and elevated caspase-3 activation, underscoring the pathogenic role of CD8^+^T cell–mediated cytotoxicity in tubular injury (171). Importantly, RTECs also modulate T cell responses through expression of the immune checkpoint ligand PD-L1. By engaging PD-1 on T cells, PD-L1 transmits inhibitory signals that establish an immunosuppressive microenvironment, partially restraining disease progression in LN (181). However, this protective mechanism may be compromised under inflammatory stimuli such as IFN-γ, which diminishes PD-L1–mediated suppression and leads to heightened T cell activation and exacerbation of immune-mediated injury. This dual function underscores the dynamic role of RTECs in balancing proinflammatory activation and immune suppression (**Figure 4B**).

B cells play a central pathogenic role in LN. Studies have shown that B cell-deficient lupus-prone mice are protected against nephritis, whereas passive transfer of autoantibodies derived from lupus models into wild-type mice can induce lupus-like kidney disease (182, 183). These findings underscore the critical role of B cells and the autoantibodies they produce in the pathogenesis of LN. B cell activating factor (BAFF) is considered a key cytokine essential for B cell survival and maturation (184). Recognized as a growth and differentiation factor for B cells, BAFF supports the survival of autoreactive B cells and enables them to escape peripheral tolerance (144, 185–187). BAFF is also regarded as a crucial cytokine in LN (144). In LN, BAFF promotes the further differentiation of B cells located in the renal interstitial space of patients (137). Notably, RTECs have been identified as an important source of BAFF (184). In lupus-prone MRL-Faslpr mice and renal biopsy samples from LN patients, tubular expression of BAFF correlates with disease activity (188). Experimental evidence also shows that the interaction between BAFF and its receptor (BAFF-R) induces the production of colony-stimulating factor 1 (CSF-1), which in turn stimulates further expression of BAFF. Additionally, in CSF-1–pretreated RTECs, BAFF stimulation has been shown to enhance cytotoxicity (188). These complex BAFF-dependent signaling pathways in RTECs may therefore contribute to the tubular cell death and atrophy observed in LN (189). Beyond modulating B cell function through BAFF signaling, B cells themselves can regulate the inflammatory response of tubular epithelial cells via co-stimulatory molecule expression. For example, B cell-expressed CD40L can engage CD40 on RTECs and myeloid cells, enhancing the pro-inflammatory activation of these innate cells and further exacerbating inflammation in the interstitial and glomerular microenvironments (164). The CD40/CD40L axis plays a specific role in B cell biology (190), including promoting B cell activation, proliferation, survival, class switching, germinal center formation, and memory B cell development (191). Accordingly, blocking this pathway has been shown to suppress disease progression in rodent models of systemic lupus nephritis with severe impairment of B cell tolerance (192, 193).

Collectively, renal structural cells such as podocytes and tubular epithelial cells function not only as passive targets but also as immunologically active participants within the inflammatory milieu of LN (194).

(A)TEC in the innate immunity. OPN on RTECs correlates with monocyte infiltration and tubular injury. In LN, monocyte-RTEC interactions via CD40-CD40L activate inflammatory pathways; RTEC PD-L1 suppresses monocyte overactivation via PD-1. CD11c^+^macrophage infiltration correlates with RTEC damage severity; *in vitro*, rIL-22-stimulated RTECs secrete CCL2/CXCL10/pSTAT3 to recruit macrophages. RTEC-derived CXCL10 recruits CXCR3^+^CD11c^+^macrophages, whose IL-6 induces fibronectin-mediated RTEC shedding/apoptosis. Macrophage-driven IL-6 further promotes RTEC apoptosis, amplifying tubular injury. RTECs recruit pDCs via TNF-α-induced chemerin-ChemR23 axis in LN. Tubulointerstitial DC3 subsets express high CXCL16/CCL17 and enhance immune priming.(B)TEC in the adaptive immunity. IFN-γ/TNF-α stimulation upregulates CXCL10 and CXCR3 expression in tubular epithelial cells at both mRNA and protein levels. In LN kidneys, injured iPTECs overexpress proinflammatory mediators, promoting blood-derived DC3 recruitment to renal tissues. Renal DC3s reprogram into proinflammatory phenotypes within the tubular injury microenvironment, driving Th1/Th17 adaptive immunity. Amplified crosstalk between Th1/Th17 and DC3s forms a proinflammatory feedback loop, synergistically aggravating renal parenchymal damage. Injured PTECs upregulate MHC-II and co-stimulatory molecules to activate CD4^+^T cells via antigen presentation. RTECs activate CD8^+^T cells through cross-presentation, inducing GzmB, IL-17A, and IFN-γ release. Direct CD8^+^T cell-RTEC contact enhances caspase-3-mediated apoptosis and tubulointerstitial inflammation.PD-L1 overexpression on RTECs suppresses T cell activation via PD-1, establishing an immunosuppressive microenvironment that counterbalances inflammatory injury.

## 6 New therapeutic targets and intervention strategies

### 6.1 Targeting renal epithelial cells

#### 6.1.1 Podocytes

Podocyte injury is a central pathological event in LN that leads to glomerular filtration barrier disruption and proteinuria (195). In recent years, multiple studies have identified several key signaling pathways and inflammatory mechanisms that regulate podocyte function, suggesting these as important targets for therapeutic intervention in LN.

A20–UCH-L1–NF-κB axis. A20, also known as tumor necrosis factor alpha-induced protein 3 (TNFAIP3), is a cytoplasmic protein that functions by inhibiting inflammation and immune responses.UCH-L1, a member of the deubiquitinating enzyme family, can catalyze the hydrolysis of Lys48-linked ubiquitin chains (196), and its expression is significantly upregulated in LN podocytes (197), resulting in podocyte injury. A20 maintains podocyte structural integrity and alleviates LN progression by suppressing NF-κB signaling, downregulating UCH-L1 expression, and reducing ubiquitin accumulation (198).

Notch1–NLRP3 pathway. Notch1 signaling is abnormally activated in LN podocytes and can induce NLRP3 inflammasome activation, triggering the release of proinflammatory cytokines and cellular injury. The γ-secretase inhibitor DAPT can effectively suppress NLRP3 activation by blocking the Notch1 pathway (195), thereby improving tissue pathology, suggesting this axis as a potential therapeutic target. Considering the complexity of intracellular signaling networks, targeting upstream regulators of inflammasome activation may represent a more effective strategy to prevent podocyte injury in LN (195). Nevertheless, the study has notable limitations, particularly the absence of validation using podocyte-specific conditional knockout models (195). Thus, the proposed mechanism warrants further investigation in rigorous animal models and clinical settings.

miR-155–SOCS1–JAK1/STAT1 pathway. In both LN patients and animal models, miR-155 is significantly upregulated and positively correlates with disease activity.SOCS1 is an important negative feedback regulator in the JAK/STAT signaling pathway, modulating inflammatory responses by inhibiting JAK1 activity and regulating STAT1 phosphorylation (199).Studies have shown that miR-155 promotes inflammatory signaling by downregulating SOCS1, thereby relieving its inhibition of the JAK1/STAT1 pathway, which enhances M1 macrophage polarization and indirectly exacerbates podocyte injury. *In vivo* experiments confirm that silencing miR-155 significantly reduces the proportion of M1 macrophages and alleviates podocyte structural damage and renal inflammation. *In vitro* studies further demonstrate that miR-155 overexpression not only promotes M1 polarization but also directly induces podocyte apoptosis (200). These findings suggest that miR-155 plays a critical role in podocyte injury and immune dysregulation by modulating the SOCS1/JAK1–STAT1 pathway, offering a novel molecular target for LN therapy (200). Although these findings provide a scientific rationale for targeting miR-155, large-scale clinical investigations remain scarce. Future multicenter clinical trials are warranted to validate the translational potential of this pathway in LN management (200).

NLRP3 Inflammasome. The NLRP3 (NOD-, LRR-, and pyrin domain–containing protein 3) inflammasome, a member of the NOD-like receptor (NLR) family, plays a pivotal role in sterile inflammation (201, 202). In LN, aberrant activation of NLRP3 has been identified in both podocytes and macrophages, correlating strongly with disease activity (203). Excessive activation of the NLRP3 inflammasome promotes the release of proinflammatory cytokines such as IL-1β and IL-18, thereby amplifying local glomerular inflammation, accelerating renal fibrosis, and contributing to podocyte injury (204).

In podocytes, activation of the NLRP3 inflammasome suppresses the expression of the key slit diaphragm protein nephrin, thereby disrupting the filtration barrier and inducing proteinuria. Experimental studies demonstrated that in LN patients and lupus-prone mouse models, the selective NLRP3 inhibitor MCC950 effectively ameliorated proteinuria, renal histopathological injury, and podocyte foot process effacement (95), underscoring its therapeutic potential in podocyte protection. Moreover, fibroblast growth factor 21 (FGF21) restored podocyte function by upregulating Irgm1, inhibiting NLRP3 inflammasome activity, and reducing the expression of pro-inflammatory mediators such as IL-1β and Caspase-1 (205). Emerging evidence also suggests that metabolic regulation may exert renoprotective effects through modulation of the NLRP3 pathway. In renal tissues from LN patients and nephritic MRL/lpr mice, the sodium-glucose cotransporter 2 (SGLT2) inhibitor empagliflozin attenuated proteinuria by enhancing autophagy to preserve cellular homeostasis and suppressing NLRP3 inflammasome activation, highlighting the need for further investigation into the renoprotective mechanisms of SGLT2 inhibitors in LN (206). Recent clinical trials(NCT05748925)further demonstrated that the addition of empagliflozin to standard therapy significantly reduced proteinuria in LN patients, suggesting its clinical potential (207). Thus, large-scale clinical trials are warranted to validate the renoprotective role of SGLT2 inhibitors in LN. Collectively, both direct and indirect NLRP3 inhibitors hold promise as future therapeutic strategies for LN; however, current evidence remains scarce and is largely limited to animal models. Beyond pharmacological interventions, gene-editing technologies also offer novel therapeutic avenues (208). A recent study by Xu et al. employed CRISPR/Cas9, a third-generation gene-editing tool, to directly disrupt NLRP3 in macrophages, thereby ameliorating various inflammatory conditions. Deletion of NLRP3 inhibited inflammasome activation *in vitro* and *in vivo*, demonstrating therapeutic promise for NLRP3-dependent inflammatory diseases (209).

#### 6.1.2 Renal tubular epithelial cells

In LN, RTECs serve not only as targets of immune-mediated injury but also as active contributors to the amplification of inflammation and the progression of fibrosis. Although glomerular lesions have been widely investigated, tubulointerstitial damage—more strongly correlated with adverse clinical outcomes—has historically received insufficient attention (210). Through a variety of signaling pathways, RTECs orchestrate local inflammatory cascades and have emerged as promising targets for therapeutic intervention.

Interferon Signaling and the Immunoproteasome. RTECs are highly responsive to IFN-α signaling, which promotes antigen presentation, immune activation, and inflammatory cytokine release (211). Activation of this pathway induces the expression of immunoproteasome subunits, thereby intensifying tubulointerstitial inflammation. Notably, inhibition of the type I interferon axis or its downstream immunoproteasome components has been shown to significantly mitigate tubular injury in experimental LN models (211).

mTOR Signaling Pathway. The mammalian target of rapamycin (mTOR) pathway is a central regulator of cell growth, metabolism, and immune equilibrium, comprising two functionally distinct complexes: mTOR complex 1 (mTORC1) and mTOR complex 2 (mTORC2) (212). Among them, mTORC1 drives tubulointerstitial remodeling by modulating cellular proliferation and protein biosynthesis and is increasingly recognized as a critical molecular mechanism underlying both SLE and its renal manifestation, LN (213). In LN patients, RTECs display persistent activation of both mTORC1 and mTORC2, with aberrant mTORC1 activation strongly linked to tubular injury and inflammatory amplification. Pharmacological inhibition of mTORC1 with rapamycin effectively suppresses this signaling pathway, reduces immune complex accumulation, and ameliorates tubular damage, offering a promising alternative for LN patients refractory to standard therapies (100). Notably, rapamycin, a selective mTORC1 inhibitor, effectively suppresses pathway activity, reduces renal immune complex deposition, alleviates tubular injury, and ameliorates disease manifestations while prolonging survival in lupus-prone mice (209, 214, 215). Furthermore, clinical studies have shown that long-term rapamycin treatment demonstrates acceptable tolerability and therapeutic efficacy in some patients with proliferative LN (100, 216), thereby highlighting mTORC1 as a promising target for the development of novel therapeutic strategies.

Vitamin D Receptor (VDR)–NLRP3 Inflammasome Axis. The vitamin D receptor (VDR) signaling pathway plays a multifaceted immunoregulatory role in controlling inflammation, including suppression of NF-κB activation, enhancement of anti-inflammatory cytokine production, and regulation of T cell differentiation (217, 218). In LN, VDR expression is markedly reduced in renal tissue and inversely correlates with disease activity and severity, highlighting its potential protective role (219). The VDR agonist paricalcitol mitigates tubulointerstitial injury through inhibition of NF-κB–driven NLRP3 inflammasome activation and attenuation of renal tubular epithelial cell (RTEC) apoptosis (220). These findings not only underscore the anti-inflammatory effects of VDR in LN but also highlight its potential as a therapeutic target. Mechanistically, nuclear-localized VDR can directly interact with NLRP3, thereby interfering with inflammasome assembly. Notably, NLRP3 activation is a critical mediator of RTEC pyroptosis, and its inhibition contributes to the preservation of tubular epithelial homeostasis. Similarly, the natural compound piperine has been reported to significantly attenuate RTEC pyroptosis and renal tissue injury through blockade of NLRP3 activation, offering a promising therapeutic strategy for LN management (221).

TGF-β–Senescence and Fibrosis Pathway. Transforming growth factor-β (TGF-β) promotes tubulointerstitial fibrosis in LN by inducing RTEC senescence and activating fibroblasts (222). One of its mechanisms is upregulation of the cyclin-dependent kinase inhibitor p15^INK4B^, which induces G1 phase cell cycle arrest, thereby inhibiting cell proliferation and driving RTECs into a senescent state. In addition, TGF-β can induce senescence-related phenotypes in various cell types, thereby contributing to persistent inflammation and tissue remodeling. TGF-β induces or accelerates cellular senescence and associated phenotypes by upregulating p15^INK4B^ and causing G1 cell cycle arrest (186, 223).

Fisetin (3,3′,4′,7-tetrahydroxyflavone) is a natural flavonoid senolytic agent found in various fruits and vegetables (224), and has been shown to effectively clear senescent cells (225, 226). It alleviates chronic inflammation and fibrosis by inducing apoptosis in senescent RTECs (224, 227, 228). Further studies have demonstrated that fisetin selectively eliminates senescent RTECs and attenuates TGF-β–driven interstitial fibrosis via inhibition of anti-apoptotic signaling pathways such as PI3K/AKT/mTOR, in a Smad-independent manner. These findings suggest that TGF-β–induced RTEC senescence plays a key role in LN-associated fibrosis, and senolytic therapies represented by fisetin may serve as a potential intervention to delay structural kidney damage (222). Notably, the PI3K/AKT/mTOR signaling pathway functions not only as a downstream effector of fisetin but also as a pivotal driver of LN pathogenesis in murine models. Aberrant activation of this pathway amplifies inflammatory and fibrotic responses. Thus, assessing PI3K/AKT/mTOR activation may yield novel insights into disease mechanisms and facilitate the development of more personalized and less toxic therapeutic strategies for LN (209).

### 6.2 Targeting immune cells

Renal epithelial cells are primary targets of immune-mediated injury in LN. Additionally, interactions between podocytes and infiltrating immune cells aggravate tissue damage, highlighting immune cells themselves as critical therapeutic targets.

#### 6.2.1 T cells

T cells play a pivotal immunoregulatory role in LN pathogenesis and represent a major focus in the development of targeted therapies. While conventional treatment approaches—including nonsteroidal anti-inflammatory drugs, glucocorticoids, and immunosuppressants (229)—remain the standard of care, their considerable toxicity and high relapse rates have driven efforts to develop more selective and less toxic immunomodulatory strategies (230).

Among numerous potential therapeutic strategies, the immunomodulatory properties of natural products have garnered significant attention. Cordyceps sinensis polysaccharide (WCP), a bioactive complex derived from the parasitic relationship between Cordyceps sinensis fungus and Lepidoptera larvae, has been shown to exert immunoregulatory effects. Previous studies indicate that Cordyceps sinensis possesses therapeutic efficacy in LN by modulating immune responses (231). Mechanistically, WCP inhibits key signaling pathways including IL-12–STAT4, IFN-γ–STAT1, and PI3K–AKT, thereby blocking Th1 cell differentiation and attenuating inflammation. Concurrently, WCP suppresses the TLR4–MyD88–MAPK signaling cascade, leading to decreased chemokine expression and reduced T cell recruitment into the kidney, ultimately conferring renal protection (232).

In addition to Th1 cells, Th17 cells are critically involved in the pathogenesis of LN. Th17-derived IL-17 activates multiple pro-inflammatory and pro-fibrotic pathways, contributing to renal dysfunction and disease progression. Therapeutic approaches targeting the Th17 axis are rapidly expanding. For instance, CaMK4 inhibitors (e.g., KN-93) can restore the Treg/Th17 balance, reduce IL-17 secretion, and improve renal function, as evidenced by reduced proteinuria (36), thereby emerging as a potential therapeutic strategy in LN. Moreover, monoclonal antibodies against IL-17A (e.g., secukinumab) or RORγt inhibitors (e.g., α-mangostin) suppress IL-17 signaling and alleviate T cell–mediated renal injury. These therapeutic approaches are currently under clinical investigation to assess their efficacy, safety, and tolerability in patients with active lupus nephritis (92). The dysregulation of the Treg/Th17 balance is a key mechanism underlying LN development. Tregs are essential for maintaining immune tolerance and suppressing autoimmunity, while Th17 cells contribute to inflammation and fibrosis through IL-17 secretion (233, 234). Multiple studies have reported a common Treg/Th17 imbalance in LN patients. Low-dose interleukin-2 (IL-2) therapy selectively expands Treg populations, significantly ameliorating renal pathology and inducing disease remission, with encouraging results from several clinical trials (233, 234). Additionally, novel chimeric antigen receptor regulatory T cell (CAR-Treg) therapies are under investigation to enhance Treg function and restore immune homeostasis, offering promising precision immunotherapeutic options for LN (235). Meanwhile, mesenchymal stem cells (MSCs), known for their dual roles in immunomodulation and tissue repair, represent another promising strategy for T cell-targeted treatment. Studies demonstrate that transplantation of umbilical cord-derived MSCs can increase Treg numbers, suppress Th17 responses (236–238), and modulate the balance of TGF-β and TNF-α, thereby improving the inflammatory microenvironment within the kidney.

Beyond the development of novel therapies, optimizing existing drugs offers additional strategies for T cell-targeted treatment. Voclosporin (VOC), a next-generation calcineurin inhibitor, reduces proteinuria by inhibiting T cell activation and, in part, through non-immune mechanisms. In phase III clinical trials(NCT02141672), the combination of VOC with mycophenolate mofetil (MMF) and low-dose glucocorticoids (GC) achieved superior renal response rates and facilitated glucocorticoid dose reduction, leading to its approval by the FDA as the first oral therapy for lupus nephritis (14, 239, 240). Interleukin-6 (IL-6), a critical mediator of T cell activation and pro-inflammatory responses, also plays a pivotal role in LN pathogenesis. Elevated IL-6 levels in systemic lupus erythematosus patients have been associated with increased disease activity (241). Tocilizumab, a humanized monoclonal antibody targeting the IL-6 receptor, significantly reduces CD4^+^T cell activation (242) and modulates multiple immune pathways involved in LN pathology. This agent is emerging as a valuable adjunct to T cell-directed therapies.

#### 6.2.2 B cells

B cells are central contributors to the pathogenesis of LN and constitute a key therapeutic target. Their survival and maturation rely on signaling mediated by surface molecules such as BAFF, CD19, and CD20 (243), making these molecules attractive targets for monoclonal antibody–based therapies.

Rituximab is a classic Type I anti-CD20 monoclonal antibody (RTX) that reduces the generation of autoantibodies by depleting CD20^+^B cells, thereby inhibiting immune complex-mediated renal injury (39, 244). *In vitro* studies have demonstrated that RTX exerts its effects through four distinct mechanisms: in the presence of FcγR-bearing cells, it induces apoptosis, complement-dependent cytotoxicity (CDC), antibody-dependent cellular cytotoxicity (ADCC), and antibody-dependent cellular phagocytosis (ADCP) (245–247). However, these findings have not convinced all researchers, prompting the re-assessment of RTX therapy (NCT01773616) in the more controlled, randomized RITUXILUP study, which investigated rituximab and mycophenolate mofetil without oral steroids for lupus nephritis treatment. Furthermore, observational studies and real-world data have shown promising results in refractory or relapsing LN patients, with an overall response rate of 50%-80%. Based on this evidence, the KDIGO 2024 guidelines have recommended rituximab as an option for patients with insufficient response to initial treatment, rather than as a first-line therapy (240). Obinutuzumab, a second-generation CD20 antibody, has a modified Fc region with enhanced glycosylation, increasing its affinity for FcγRIII and significantly improving ADCC and ADCP effects (248, 249). In refractory LN, obinutuzumab has demonstrated superior B-cell depletion efficacy and higher clinical remission rates (250). Recently, in a phase II, double-blind, randomized controlled trial (NCT02550652), obinutuzumab, in combination with mycophenolate mofetil and corticosteroids, showed superior outcomes compared to placebo (39). Moreover, obinutuzumab has shown favorable safety, with guidelines suggesting that it may overcome some limitations of rituximab by providing more effective and durable B-cell depletion. However, the long-term efficacy and safety of obinutuzumab require further validation through phase III clinical trials to determine its precise role in the therapeutic landscape of lupus nephritis (240).

BAFF, a member of the TNF superfamily secreted by myeloid cells, facilitates the transition of immature to mature B cells and promotes plasma cell survival and sustained autoantibody production (251). Belimumab, a humanized IgG1λ monoclonal antibody, selectively neutralizes soluble BAFF and blocks receptor engagement, thereby reducing CD20^+^B cells and plasma cells, leading to diminished autoantibody titers (39). In the phase III BLISS-LN trial (NCT01639339), the addition of belimumab to standard therapy significantly increased the proportion of patients achieving primary renal efficacy response at two years compared with placebo plus standard therapy. Multiple clinical trials have demonstrated that belimumab confers significant clinical benefits in LN, including higher remission rates, delayed progression of renal dysfunction, and reduced relapse rates, while maintaining a favorable safety profile. On this basis, the KDIGO 2024 guidelines recommend belimumab as a first-line therapeutic option (240). Its efficacy is especially pronounced in patients receiving concomitant MMF therapy (240).

Additionally, phosphoinositide 3-kinase alpha (PI3Kα), a lipid kinase expressed in multiple tissues, plays a crucial role in B cell activation, metabolism, and migration (252). Pharmacological inhibition of PI3Kα has been shown to suppress proinflammatory cytokine secretion, reduce B cell–mediated immune responses, attenuate autoantibody production, and mitigate glomerular complement deposition (253). These findings highlight PI3Kα as a promising therapeutic target in LN.

#### 6.2.3 Therapeutic targeting of costimulatory pathways

Effective T cell activation requires not only antigen recognition via the MHC–antigen complex (signal one) but also a second, costimulatory signal. Costimulatory molecules CD80 and CD86 on APCs or B cells engage CD28 on naïve T cells, promoting their activation and clonal expansion. Cytotoxic T lymphocyte–associated antigen 4 (CTLA4), expressed on activated T cells, competes with CD28 for binding to CD80/CD86, thereby dampening the T cell response. Abatacept, a CTLA4–Ig fusion protein, acts as a selective costimulatory modulator by binding CD80/CD86 and inhibiting CD28-mediated T cell activation (14).

Another critical costimulatory pathway involves CD40 and its ligand CD40L. CD40L is predominantly expressed on activated T cells, while CD40 is present on B cells, dendritic cells, and intrinsic renal cells such as proximal tubular epithelial cells (254, 255). Activation of this axis facilitates autoantibody production, promotes their deposition in renal tissue, and amplifies local inflammation through enhanced B cell expansion and activation of myeloid and epithelial cells. These effects collectively exacerbate glomerular and tubulointerstitial injury in LN (164). Preclinical studies have shown marked upregulation of CD40 expression in the kidneys of LN models, and blockade of the CD40–CD40L interaction significantly attenuates renal inflammation and immunopathology (164). Collectively, these findings underscore the therapeutic potential of costimulatory blockade in LN. Targeting early T cell–costimulatory interactions offers a promising strategy to suppress aberrant immune activation and prevent immune-mediated renal damage.

## 7 Future perspectives

Although emerging therapies demonstrate notable short-term benefits and steroid-sparing effects, their long-term safety remains to be carefully assessed. For instance, voclosporin (VOC) may aggravate hypertension and reduce glomerular filtration rate (GFR), whereas belimumab has been associated with neuropsychiatric adverse events such as insomnia and anxiety (256–259). Similarly, the risk of chronic nephrotoxicity from calcineurin inhibitors (CNIs) and immunosuppression-related infections induced by biologics highlights the necessity of continuous monitoring of long-term safety profiles (260–263). These challenges underscore the urgent need to move toward precision and individualized therapeutic strategies. By integrating molecular subtyping and biomarkers (e.g., anti-dsDNA antibodies and renal transcriptomic signatures) with artificial intelligence–based algorithms, it may become possible to more accurately stratify patients and optimize therapeutic decision-making (264). In parallel, emerging technologies are opening new avenues for mechanistic exploration. Single-cell RNA sequencing enables the mapping of interaction networks between immune cells and renal epithelial cells, while urinary proteomics provides promising opportunities for noninvasive disease monitoring and patient stratification (42). Building upon these advances, multitarget combination strategies may represent a pivotal approach to balancing efficacy and safety. Combinations such as belimumab with VOC, or with complement inhibitors, hold potential to broaden the therapeutic landscape (265). Collectively, these developments not only address the limitations of single-target therapies but also pave the way for transitioning lupus nephritis management from conventional immunosuppression toward precision immunomodulation.

## 8 Conclusion

LN represents a severe manifestation of systemic lupus erythematosus, wherein intricate crosstalk between renal epithelial cells and immune cells plays a pivotal role in disease pathogenesis. Podocytes, as integral components of the glomerular filtration barrier, are susceptible to injury by autoantibodies, immune complexes, and inflammatory mediators. Beyond serving as passive targets, podocytes actively participate in immune regulation by expressing molecules such as MHC and costimulatory proteins, thereby contributing to antigen presentation and activation of T and B lymphocytes, further amplifying local immune responses and tissue damage. RTECs, similarly, are not merely victims of immune attack. They actively secrete cytokines and chemokines that recruit and modulate immune cells and undergo EMT, promoting tubulointerstitial fibrosis. On the immune side, dysregulation of T cell subsets—particularly Th17 cells—drives podocyte injury via proinflammatory cytokines, while antigen presentation interactions between T cells and RTECs exacerbate inflammation. B cells contribute through the production of pathogenic autoantibodies and engage in direct crosstalk with RTECs to potentiate immune-mediated injury. Innate immune cells, including macrophages, dendritic cells, and neutrophils, further aggravate renal damage through cytokine release, antigen presentation, and oxidative stress. These cell populations form a complex and dynamic communication network, mediated by cytokines, chemokines, and cell-surface interactions. Elucidating these intercellular mechanisms is essential for identifying novel therapeutic targets and developing more precise and effective treatment strategies aimed at improving long-term outcomes in patients with LN.
